# Uptake of health services for common mental disorders by first-generation Turkish and Moroccan migrants in the Netherlands

**DOI:** 10.1186/1471-2458-9-307

**Published:** 2009-08-23

**Authors:** Thijs Fassaert, Matty AS de Wit, Arnoud P Verhoeff, Wilco C Tuinebreijer, Wim HM Gorissen, Aartjan TF Beekman, Jack Dekker

**Affiliations:** 1 Department of Epidemiology Documentation & Health Promotion, Municipal Health Service, Amsterdam, the Netherlands; 2 Department of Social Medicine, Academic Medical Centre (AMC/UvA), Amsterdam, the Netherlands; 3 Department of Social and Behavioural Sciences, University of Amsterdam, Amsterdam, the Netherlands; 4 Department of Public Mental Health Care, Municipal Health Service, Amsterdam, the Netherlands; 5 Arkin Academy, Arkin Mental Health Institute, Amsterdam, the Netherlands; 6 Department of Psychiatry, VU University, Amsterdam, the Netherlands; 7 GGZ inGeest, Amsterdam, the Netherlands; 8 Department of Clinical Psychology, VU University Amsterdam, the Netherlands; 9 Research Department, Arkin Mental Health Institute, Amsterdam, the Netherlands

## Abstract

**Background:**

Migration and ethnic minority status have been associated with higher occurrence of common mental disorders (CMD), while mental health care utilisation by non-Western migrants has been reported to be low compared to the general population in Western host countries. Still, the evidence-base for this is poor. This study evaluates uptake of mental health services for CMD and psychological distress among first-generation non-Western migrants in Amsterdam, the Netherlands.

**Methods:**

A population-based survey. First generation non-Western migrants and ethnic Dutch respondents (N = 580) participated in structured interviews in their own languages. The interview included the Composite International Diagnostic Interview (CIDI) and the Kessler psychological distress scale (K10). Uptake of services was measured by self-report. Data were analysed using weighting techniques and multivariate logistic regression.

**Results:**

Of subjects with a CMD during six months preceding the interview, 50.9% reported care for mental problems in that period; 35.0% contacted specialised services. In relation to CMD, ethnic groups were equally likely to access specialised mental health services. In relation to psychological distress, however, Moroccan migrants reported less uptake of primary care services (OR = 0.37; 95% CI = 0.15 to 0.88).

**Conclusion:**

About half of the ethnic Dutch, Turkish and Moroccan population in Amsterdam with CMD contact mental health services. Since the primary purpose of specialised mental health services is to treat "cases", this study provides strong indications for equal access to specialised care for these ethnic groups. The purpose of primary care services is however to treat psychological distress, so that access appears to be lower among Moroccan migrants.

## Background

Equal access to care is a key-characteristic of quality of care and a necessary – though not sufficient – condition for equal opportunity to health [1,2]. Within Western European countries, where substantial and still increasing parts of the general population consist of non-Western migrant populations, equal access to care is also an important subject from a more political and moral point of view [3,4]. A growing proportion of these migrants have Muslim-religious backgrounds, and current political and social circumstances – by some characterised as "Islamophobia" – have been argued to contribute to the marginalisation of this religious minority [5,6]. Both migration and marginalisation have been suggested as possible risk factors for (mental) health problems [5-8], which poses challenges for research in determining whether current uptake of health services by non-Western migrants adequately meets their needs. As far as mental health problems are concerned, there are indications that this is not the case [9]. The present study focuses on uptake of services for anxiety and depression, or common mental disorders (CMD).

Apart from factors like gender, age (predisposing factors), income and education (enabling factors), health care utilisation is best predicted by need factors [10]. Considering the organisation of the Dutch health care system, two types of mental health care need are of interest. This is because Dutch health care has a 'referral' system, meaning that patients cannot directly consult a medical specialist, but have to visit a general practitioner (GP) first. GPs subsequently acts as gatekeepers (i.e. have to recognise a potential CMD illness and may subsequently refer to specialised mental health care) [11]. Following from this, primary care services in the Netherlands are typically those services to which patients are self-referred when they have a subjective need [12]. Instead, the traditional purpose of specialised mental health services is the diagnosis and treatment of (common) mental disorders. The need for care is therefore determined more objectively, by the presence of mental illness (or 'caseness') [13].

Studying equity in access to mental health services for CMD thus involves information about objective and subjective need in relation to CMD. Gathering data about the prevalence of mental disorders among non-Western migrants has however been shown to be difficult [14-16]. As a consequence of language problems and a lack of cross-culturally validated instruments of measurement, for example, minorities are often excluded from epidemiological studies [14,17,18]. Moreover, research into the mental health of non-Western migrants is often impeded by barriers that relate to their traditional and religious culture [19].

By way of applying an elaborate set of strategies to limit selective non-response, this study was conducted among first generation (i.e. foreign born) Turkish and Moroccan migrants in the Netherlands. Both groups belong to the largest migrant groups in Western Europe [20,21], and by far most of them have a Muslim religious background. There are very strong indications that CMD are more prevalent among Turkish and Moroccan migrants [15,22-24]. While they are well represented in Dutch general practice [3,25], it is unclear if this is for mental health problems. Many of the factors that act as barriers in epidemiological research among ethnic groups also serve as obstacles in the help-seeking process. For example, Turkish and Moroccan migrants are known to be reluctant in reporting mental health problems, and to focus on somatic symptoms instead [15]. This reluctance and somatisation, but also problems in doctor-patient communication and low socioeconomic status, are likely to diminish the probability that mental health problems are reported or identified as such during a consultation [3,26,27]. Because GPs in the Netherlands operate as gatekeepers to specialised health services, migrants are consequently believed to be underrepresented in specialised (mental) health care [3,25,28-30].

This population-based study focuses on the following questions: (a) what is the degree of uptake of primary and specialised mental health services among first-generation Turkish and Moroccan migrants and ethnic Dutch subjects with CMD, (b) is uptake of mental health services by migrants different compared to ethnic Dutch, and (c) do differences persist/appear when differences in predisposing, enabling and (objective/subjective) need factors are taken into account?

## Methods

### Procedure

The study was conducted using data from 580 subjects (304 ethnic Dutch and 276 first-generation Turkish and Moroccan migrants). These were sampled from the 2004 Amsterdam Health Monitor (AHM), carried out by the Amsterdam Municipal Health Service (GGD) in collaboration with the National Institute for Public Health and the Environment (RIVM) [31,32]. The AHM 2004 was designed to map the general health status of the Amsterdam population, aged 18 years or older, by means of a structured interview and a physical examination. A random sample was drawn from the municipal population registration, and stratified by ethnicity (Dutch, Turkish, Moroccan and other) and age groups (18–34, 35–44, 45–54, 55–64 and 65 years and older). In conformation with the definition of Statistics Netherlands, a subject was considered to be a first-generation Turkish or Moroccan migrant if that person was born in Turkey or Morocco, regardless of where his or her parents were born [33]. Likewise, respondents were considered ethnic Dutch if both parents of the respondent were born in the Netherlands.

During the AHM 2004, various measures were taken to increase the response. These included (a) an announcement of the survey by mail (in multiple languages) and local media (e.g. on a local Turkish radio station), (b) an additional reminder in the week before the data-collection commenced, (c) multiple contact attempts, (d) translation of instruments into English, Turkish and Standard Arabic, (e) the application of oral interviews as opposed to questionnaires, (f) ethnic matching of interviewers and respondents (optional), (g) employment of bi-lingual interviewers and (h) a financial incentive (15 Euros) for actual participation.

The generic AHM 2004 was followed by a 'second wave', which consisted of a structured interview that was specifically aimed at mental health, and which provided data for the present study [23]. All respondents from the first wave who consented with a second approach were asked to participate. Again, a number of precautionary measures was taken to minimise non-response. In addition to the aforementioned measures, respondents were visited at home, they could chose the location of the interview (i.e. at home or in a neutral environment), and each interview was planned between February and June, as to avoid Christmas, Ramadan and the summer holidays. All study procedures were approved by the ethical commission of the Amsterdam Academic Medical Centre.

### Response

Among ethnic Dutch, Turkish and Moroccan respondents the response rate during the AHM 2004 was 44.7% (N = 1307). Specific response rates are described in de Wit et al. [23]. Of these, 1076 subjects (82.3%) were willing to participate in any follow-up study and were therefore eligible for inclusion. The response rate for the eligible population was 67.3% (i.e. 320 ethnic Dutch, 191 Moroccan, 213 Turkish). We studied only first-generation Turkish and Moroccan migrants with complete data on all relevant variables. Our study sample thus consisted of 580 ethnic Dutch and first-generation Turkish and Moroccan migrants.

During the first wave of the AHM, response was significantly lower in the lowest age-group (18–34 years; p < 0.001), among men (39.6%) compared to women (50.4%; p < 0.001), and among Moroccans (38.8%) compared to ethnic Dutch (45.9%) and Turkish (49.6%; p < 0.001). With respect to differences in income and unemployment, only raw comparisons were made, since data were available at different levels (e.g. spendable household income per year from the municipality vs. self-reported monthly family income after tax from the AHM) [32]. Among subjects in the AHM, 38% reported a family income of €17.550, 48% reported an income between €17.550 and €41.600, and 14% had an income of at least €41.600. This is comparable to the distribution of income in the general Amsterdam population, since 31% of the population in 2004 had spendable household incomes below €15.800, 54% had incomes between €15.800 and €39.900, and 15% had an income of at least €39.900 per year. Regarding employment rates, 5% of AHM respondents reported to be jobless, while 7% of the general Amsterdam population was known to be unemployed in 2004 [32].

After the second wave de Wit et al. [23] calculated an overall response rate of 26.5%, ranging between 20.8 (Moroccans) and 30.2 (ethnic Dutch). The follow-up rate was lower among Turkish and Moroccans (62.2% and 70.5% respectively) than among ethnic Dutch (76.9%; p < 0.001), and lower among men (68.1%) than among women (73.2%; p = 0.027). However, there was no selection with respect to age (p = 0.856). Between participants and non-participants in the second wave, there were also no significant differences regarding perceived health status (p = 0.101), psychological distress (p = 0.635), general practice visits (p = 0.101) and outpatient health care utilisation (p = 0.480) in the past two months, any health care utilisation for mental health problems in the past year (p = 0.903), and current use of psychotropics (p = 0.903).

### Measures

The outcome measure for this study indicated whether or not a respondent contacted general health care for mental problems and/or specialised mental health care. For that purpose, health care utilisation was measured with an adapted version of the Trimbos/iMTA questionnaire for Costs associated with Psychiatric Illness [34]. For each type of care deliverer, respondents reported the number of contacts during the past six months and if at least one of those contacts was for mental health problems. Contact with primary care services for mental health problems was defined as at least one visit to a GP or company-doctor for mental health problems, or a visit to a first line psychologist, general social worker or social-psychiatric nurse. Specialised mental health services were defined as ambulatory mental health care, a visit to a Centre for Alcohol and Drugs, or contact with a private psychiatrist/psychotherapist.

Psychiatric morbidity (i.e. presence of CMD in the past six months) was measured with the Composite International Diagnostic Interview (CIDI) Basis Life time version 2.1 [35]. The CIDI has been translated into Dutch, Turkish and Arabic [36,37]. Only the CIDI sections for depression and anxiety were selected, because of the population-based design of the study: depression and anxiety are the most common mental disorders in the general population. Specific phobias were excluded from the interview.

While the CIDI diagnosis for a CMD was a measure of objective need, psychological distress was measured additionally as to give subjects the opportunity to also express subjective need. Psychological distress was measured using the Kessler psychological distress scale (K10) [38]. Items of the K10 measure the extent to which psychological symptoms are present (e.g. 'During the past 30 days, about how often did you feel tired out for no good reason?') with five response categories: 'none of the time', 'a little of the time', 'some of the time', 'most of the time' and 'all of the time'. The total score is the sum of all responses; scores thus range between 10 and 50. For further information about the K10 the reader is referred to the National Comorbidity Survey (NCS) website . In the present study, Cronbach's alpha was 0.93, indicating a very high internal consistency. Previous research supported the validity and reliability of the K10 as an instrument to screen for anxiety and depression among Turkish, Moroccan and ethnic Dutch subjects [39].

Finally, information was available about age and gender, type of health insurance and highest level of education attained. The latter two were included as indicators of SES. Type of health insurance used to be linked to income because, until January 2006, people with a higher income had a private insurance, as opposed to mandatory public insurance for people with lower incomes. Furthermore, almost everybody in the Netherlands has medical insurance and thus there were virtually no missing values on this variable. Both education and income are considered suitable indicators of SES and have several advantages over occupational class [40].

### Analysis

An extensive non-response analysis was done, using information on (mental) health and health care utilisation from the generic (first) wave. Differences were analysed using ANOVA, Chi^2^-tests and Fisher's exact tests. To answer the study questions, health care utilisation was analysed by calculating proportions weighed according to the distribution of age, gender and ethnic background in the Amsterdam population in 2004, thereby accounting for possible selective response in relation to these characteristics. Furthermore, multivariate logistic regression analyses were conducted, in which outcome variables indicated whether or not a subject reported primary and specialised mental health services, respectively. Odds ratios (ORs) were calculated, corrected for differences in age and gender (model 1), presence of CMD (model 2), psychological distress (model 3), and SES (model 4). Each step in the analysis was judged on its significance. Finally, we studied interaction between ethnic background and mental health characteristics to see whether there were differences in access among those who were in need vs. those who were not in need. All analyses were done in SPSS version 15.

## Results

Migrants were significantly younger than ethnic Dutch respondents (table 1). Among Moroccans, the proportion of men was significantly higher. Migrants were generally less well educated, more likely to be publicly insured and attained significantly higher scores on the K10. The prevalence of a six-month diagnosis for a mood and/or anxiety disorder was much higher among Turks [23].

**Table 1:** Sociodemographic characteristics of the study population, per ethnic group (N = 580)

	Ethnic Dutch (N = 304)	Turkish (N = 149)	Moroccan (N = 127)	p-value*
Age (mean, sd.)	53.8 (14.5)	47.8 (13.2)	50.4 (12.9)	0.000
Gender (male, %)	41.8	45.6	55.9	0.027
Education (higher than vocational, %)	80.6	43.0	41.7	0.000
Health insurance (public, %)	62.5	81.2	95.3	0.000
CIDI-diagnosis for CMD (%)	10.9	27.5	12.6	0.000
K10 (mean, sd.)	15.0 (5.2)	20.8 (8.8)	19.0 (9.5)	0.000

* differences were tested using ANOVA (means) and Chi^2^-tests (proportions)

Sd. = standard deviation

CIDI = Composite International Diagnostic Interview

CMD = common mental disorder (anxiety and/or depression)

K10 = Kessler psychological distress scale

### Uptake of services

Table 2 shows the weighted proportions for primary and specialised health services for mental health problems among subjects with CMD, showing that 50.9% reported any type of help for mental health problems in the past six months. Almost 16 percent reported only primary care services for mental problems and 35.0% reported specialised mental health care. Services utilisation for mental health problems appeared higher among migrants, although observed differences between ethnic groups were not statistically significant (Chi^2 ^= 0.533, df1 = 3, df2 = 211, p = 0.868).

**Table 2:** Mental health care utilisation by Amsterdam citizens with a mood and/or anxiety disorder in the previous 6 months, weighted for age and sex

	All *	Ethnic Dutch	Turkish	Moroccan
No mental health care	49.1	50.9	43.5	39.2
Only primary care services	15.9	15.2	18.3	19.3
Also specialised mental health care	35.0	33.9	38.2	41.5

Total	100	100	100	100

* Also weighted for ethnic background

In table 3, ORs corrected for age and sex (model 1) indicated that Turkish were more likely to contact primary care services for mental health problems than ethnic Dutch. There were no other ethnic differences. Entrance of CMD and psychological distress to the model (i.e. steps 2 and 3) both yielded highly significant steps (p ≤ 0.001). After entering CMD, ORs for Turkish and Moroccan ethnicity generally decreased, but did not indicate significant differences in care utilisation compared to ethnic Dutch. Inclusion of psychological distress, however, revealed significantly lower use of primary mental health care among Moroccans (p = 0.025). Differences in uptake between Moroccans and ethnic Dutch could not be explained by differences in SES, as step 4 in the analysis was highly insignificant. Further investigation of the results learned that there was indeed no association between uptake of primary services for mental health problems and being higher educated (OR = 0.82; 95% CI = 0.43–1.56) or having private health insurance (OR = 0.90; 95% CI = 0.47–1.73).

**Table 3:** Association between ethnic background and uptake of primary health services for mental health problems and specialised mental health services (N = 580)*

	**Turkish** **(N = 149)**	**Moroccan** **(N = 127)**		
**Primary care**	OR (95% CI)	OR (95% CI)	Chi^2 ^value step (df)	p-value step
Model 1^a^	1.83 (1.06–3.16)	0.70 (0.34–1.43)	12.63 (4)	0.013
Model 2^b^	1.14 (0.62–2.12)	0.60 (0.27–1.30)	68.92 (1)	< 0.001
Model 3^c^	0.78 (0.40–1.53)	0.37 (0.15–0.88)	12.10 (1)	0.001
Model 4 ^d^	0.85 (0.42–1.75)	0.41 (0.16–1.01)	0.52 (1)	0.770

**Specialised care**	OR (95% CI)	OR (95% CI)	Chi^2 ^value step (df)	p-value step
Model 1^a^	1.66 (0.85–3.23)	1.03 (0.47–2.25)	4.41 (4)	0.353
Model 2^b^	0.97 (0.46–2.04)	0.94 (0.40–2.19)	56.0 (1)	< 0.001
Model 3^c^	0.47 (0.20–1.10)	0.39 (0.14–1.07)	25.05 (1)	< 0.001
Model 4 ^d^	0.48 (0.19–1.17)	0.38 (0.13–1.10)	0.33 (1)	0.350

* ethnic Dutch serve as reference category

OR = odds ratio

CI = confidence interval

^a ^Model 1: ethnicity + age + sex

^b ^Model 2: ethnicity + age + sex + CIDI

^c ^Model 3: ethnicity + age + sex + CIDI + K10

^d ^Model 4: ethnicity + age + sex + CIDI + K10 + socioeconomic status (SES)

Regarding specialised mental health services, there were no differences in uptake between migrants and ethnic Dutch when taking into account differences in age and sex (model 1) or prevalence of CMD (model 2). Entering psychological distress to the model (model 3) suggested lower uptake by Turkish and Moroccan migrants, but the differences were not statistically significant. Again, SES variables did not pay a significant contribution to the model (model 4); the associations between uptake of specialised mental health services and being higher educated (OR = 0.88; 95% CI = 0.41–1.91) or having private health insurance (OR = 1.24; 95% CI = 0.54–2.85) were statistically non-significant.

Finally, there was no statistically significant interaction between ethnic background and presence of CMD, or ethnic background and psychological distress, with respect to primary mental health care utilisation. Regarding secondary mental health services there was no interaction between ethnic background and mental health status either.

## Discussion

In an increasingly multicultural society, equity in access to health services is a highly valued and essential dimension of quality of care. In that context, the aim of the present study was to focus on possible inequalities in uptake of mental health services in a population based sample of first generation Turkish and Moroccan migrants and ethnic Dutch respondents. The study investigated (a) the degree of uptake of primary (i.e. generic) and specialised mental health services among first-generation Turkish and Moroccan migrants and ethnic Dutch subjects with CMD in Amsterdam, (b) possible differences in uptake of mental health services between migrants and ethnic Dutch, and (c) the influence of predisposing (age, gender) and enabling (SES) factors on possible differences.

In our study, more than 50 percent of subjects with CMD reported uptake of care for mental health problems during the six months preceding the interview. Of those who reported care, the majority reported contact with specialised mental health care, which is in line with other studies in the general Dutch population [41,42]. Among subjects with a CMD, there were no statistically significant differences between ethnic Dutch and non-Western migrants, either Turkish or Moroccan, regarding uptake of formal mental health services. This was confirmed by the regression analyses, since (i) on average there were no differences in mental health services across migrant groups in relation to the presence of CMD, and (ii) there was no interaction between ethnic background and presence of CMD. However, the analyses also showed that migrants reported more psychological distress than did ethnic Dutch respondents. In relation to these higher subjective levels of mental illness, the uptake of mental health services was lower among (Moroccan) migrants. This difference between ethnic groups could not be explained by differences in socioeconomic status.

To understand the implications of these results, it is necessary to consider again the purposes of the different types of mental health care that were studied. Equal access to care in this study was defined as equal access to available care for equal need. With respect to specialist care, we defined need for care by the presence of mental illness (CMD), or 'caseness' [13]. Our study shows that, given the presence of a common mental disorder, Turkish and Moroccan migrants did not differ in the uptake of specialised mental health services compared to ethnic Dutch. This is in line with the recent finding that, at least in Amsterdam, migrants seem to be catching up in access to and use of outpatient mental health services [43]. Additionally, in a study that included ethnic Dutch, Turkish and Moroccan inhabitants of Rotterdam, which is another large urban area in the Netherlands, only Moroccan women were found to be underrepresented in specialised mental health care [44]. Notably, this is also the group that least often met the criteria for a mood and/or anxiety disorder [23].

The principle of *caseness *applies less to primary care services. Instead, subjective need plays a major role [12]. In that context it is noteworthy that, given a certain level of psychological distress, Moroccan migrants were less likely to report uptake of primary care for mental health problems. This is a disturbing finding, for Turkish and Moroccan migrants are both considered to be frequent visitors of general practice [3,25]. According to our study, they are however less likely to do so for mental health problems. This finding suggests differential uptake of primary mental health services.

There may be several explanations for the observation that Moroccan migrants were less likely to report uptake of primary care for mental health problems. These include stigma or taboo attached to mental health problems, disproportionate somatisation of mental health problems, or communication problems in the doctor-patient relationship at different levels [3,19,26,27]. Communication, for example, is complicated if patient and provider do not share the same linguistic skills, have different ideas about illness, or have prejudices against each other [45]. Such problems negatively affect patient satisfaction, patient compliance, perception of a good interpersonal relationship, and patient trust in the physician [46,47]. Indeed, problems in communication between ethnic minority clients and GPs have been associated with lower patient satisfaction and lower perceived quality of care [48], which decrease attendance in general practice for mental health problems. It could be that Moroccan clients in this respect differ from Turkish clients. For example, educational levels are generally somewhat higher among first-generation Turkish migrants than among Moroccan migrants. For example, illiteracy tends to occur less often among Turkish than among Moroccan first-generation migrants [49], while it is known that illiteracy is associated with ill health [50].

An additional explanation for the finding with respect to primary care may be sought in the concept of perceived need for care. Indeed, a higher perceived need for care has been associated with increased service use, better compliance with treatment, and less dropout [51,52]. Moreover, if people are convinced that problems do not require treatment, this is an important reason for them not to seek help [53,54]. So, although the term 'subjective need' would suggest differently, the experience of psychological distress does not necessarily imply that a need for mental health care is perceived. This discrepancy between symptom experience and care utilisation can be influenced by ethnic minority background, among other factors [55]. In a recent study within the same study population, it was found that in case of similar mental morbidity, perceived need for information, psychotropics, referral to specialised mental health care, or for counselling was lower among Moroccan migrants than among ethnic Dutch [56]. We recommend that future studies in this area take the possible influence of perceived need for care into account.

Various authors have argued that mental health care utilisation among Turkish and Moroccan migrants is lower than among ethnic Dutch. The observation that access of specialised mental health services was relatively equal for all three ethnic groups is therefore surprising. The limitations of the present study notwithstanding, there are reasons why this observation may nevertheless reflect the actual situation in present-day Dutch urban mental health care. First, it should be noted that most other studies addressing this issue tend to use a very different definition of equal access to care, namely equal representation of minority groups in (mental) health care compared to their representation in the general population. In contrast, we defined equal access on the basis of need factors, and we used population-based prevalence estimates to define this need. Second, health care in the Netherlands nowadays has a history of 'interculturalisation', which means that numerous efforts have been made to adapt mainstream health services to suit clients from different cultures, as opposed to the development of health services for specific ethnic groups [57]. Based on these and other recent results [43,44] one could argue that this may have had a positive effect on accessibility of specialised mental health services. At the same time, however, it should be noted that equal access – though a necessary condition for equal opportunity to health – does not guarantee equal result [58].

### Strengths and limitations

Apart from the fact that this is one of the first population-based studies among two major European migrant populations to address inequalities in mental health care for common mental disorders, additional strengths should be mentioned. For example, the study was conducted by well-trained bilingual interviewers, whose ethnic backgrounds were matched to the background of respondents. These and other measures resulted in the inclusion of respondents who were not fluent in Dutch, while most epidemiological studies in this area on beforehand exclude respondents who do not sufficiently master the dominant language(s) of the host country [14]. Also, the study was conducted in a large urban area, which makes the results potentially interesting for other urban settings.

However, there are some limitations to this study that need to be addressed as well. Firstly, despite all measures to increase response, we acknowledge that the generalisability of our results is compromised by the high non-response and incompleteness of data. Considering the efforts that have been made to limit non-response, the response rate in the present study may yet be the highest feasible response for this type of research [17]. Although non-response in the second wave appeared to be non-selective regarding mental health outcomes, and weighting techniques were used to correct for selective non-response according to demographic factors, the small sample size limited the statistical power of the analyses. Though we acknowledge this is partially related to the deletion of cases with missing data, we refrained from data imputation techniques for two reasons. Firstly, it is known that imputation can distort coefficients of association and correlation relating variables. Secondly, the number of cases with missing data was relatively small. As a rule of thumb, if a variable has more than 5% missing values, cases are not deleted [59]. Educational level (8.6%) was the only variable that – barely – exceeded this level.

A second limitation is that health care uptake was measured by way of self-report, which may have resulted in response bias. Still, though this may have influenced the estimates of uptake of mental health services, self-report measures are considered suitable for studying ethnic *differences *in care utilisation [60,61].

Third, the results of the statistical analyses may have been sensitive to model specification, which in this case could have resulted from the failure to include other important variables. For that matter, we fully acknowledge that Anderson's behavioural model specifies other relevant variables that could have been acting as confounders. Examples are marital status, health beliefs, and acculturation (predisposing), social support (enabling), and somatic comorbidity (need). Inclusion of these variables would have probably resulted in more accurate results. At the same time, however, the limited sample size urged us to be very conservative in the number of covariates that we could include, and to only include the most relevant information.

Finally, SES was indicated only by two rough measures of education and income, while the concept of SES is much broader [1]. Given the fact that most non-Western migrants have a relatively low SES, the influence of socioeconomic position was very difficult to study in this particular sample. It is strongly recommended that future studies make efforts to further disentangle ethnic and socioeconomic influences in the context of mental health services research.

## Conclusion

In summary, more than half of people with a CMD reported uptake of help for mental health problems. Of them, the majority contacted specialised mental health services. There was equal uptake of specialised mental health services across ethnic groups, given that the purpose of specialised mental health services is to treat "cases". Uptake of primary care services, however, is generally guided by self-referral. In that context, there was lower uptake of primary care services by (Moroccan) migrants in relation to the amount of psychological distress.

## Competing interests

The authors declare that they have no competing interests.

## Authors' contributions

TF and MASW formulated the research question. MASW, WCT, APV, WHMG, ATFB and JD participated in the design of the study. TF performed the statistical analyses and drafted the manuscript together with MASW. WCT, APV, WHMG, ATFB and JD contributed to the manuscript with their interpretation of results and comments on earlier drafts. All authors read and approved the final manuscript.

## Pre-publication history

The pre-publication history for this paper can be accessed here:


